# Thrombocytopenia in dengue infection: mechanisms and a potential application

**DOI:** 10.1017/erm.2024.18

**Published:** 2024-10-14

**Authors:** Ahmad Suhail Khazali, Waqiyuddin Hilmi Hadrawi, Fatimah Ibrahim, Shatrah Othman, Nurshamimi Nor Rashid

**Affiliations:** 1 Faculty of Applied Sciences, Universiti Teknologi MARA (UiTM) Cawangan Perlis, Arau, Perlis, Malaysia; 2 Department of Molecular Medicine, Faculty of Medicine, Universiti Malaya, Kuala Lumpur, Malaysia; 3 Department of Biomedical Engineering, Faculty of Engineering, Universiti Malaya, Kuala Lumpur, Malaysia; 4 Center for Innovation in Medical Engineering (CIME), Faculty of Engineering, Universiti Malaya, Kuala Lumpur, Malaysia

**Keywords:** immature platelet fraction, megakaryopoiesis, platelet, thrombocytopenia, thrombopoiesis

## Abstract

Thrombocytopenia is a common symptom and one of the warning signs of dengue virus (DENV) infection. Platelet depletion is critical as it may lead to other severe dengue symptoms. Understanding the molecular events of this condition during dengue infection is challenging because of the multifaceted factors involved in DENV infection and the dynamics of the disease progression. Platelet levels depend on the balance between platelet production and platelet consumption or clearance. Megakaryopoiesis and thrombopoiesis, two interdependent processes in platelet production, are hampered during dengue infection. Conversely, platelet elimination via platelet activation, apoptosis and clearance processes are elevated. Together, these anomalies contribute to thrombocytopenia in dengue patients. Targeting the molecular events of dengue-mediated thrombocytopenia shows great potential but still requires further investigation. Nonetheless, the application of new knowledge in this field, such as immature platelet fraction analysis, may facilitate physicians in monitoring the progression of the disease.

## Introduction

**Figure 1.**
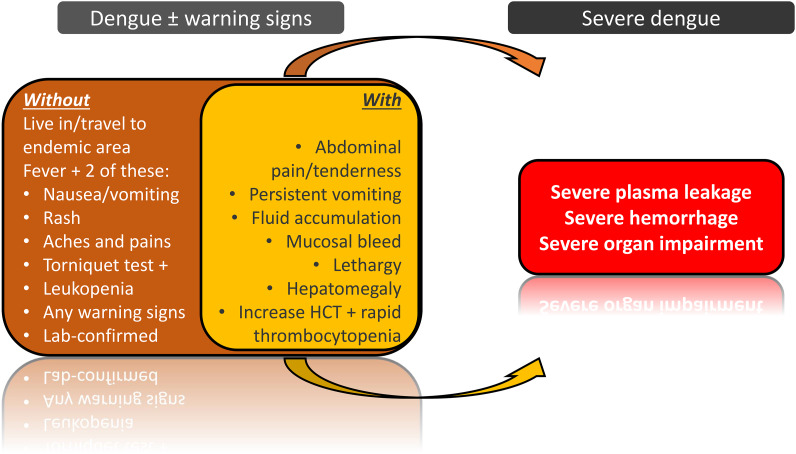
Classification of dengue by the WHO. This information is based on 2009 WHO guideline (Ref. 1). The present work focuses on one of the symptoms of dengue with warning signs which is thrombocytopenia.

Dengue is a mosquito-borne disease common in tropical and subtropical countries. Although dengue infection is usually non-life threatening, progression into the severe form of the disease could be fatal because of serious complications associated with severe dengue. In 2009, the World Health Organization (WHO) revised the characterization of dengue infection into two main categories: non-severe dengue and severe dengue (Ref. 1). The non-severe dengue is further divided into two subcategories: dengue without warning signs and dengue with warning signs. This revised characterization aims to facilitate clinicians in diagnosing patients with warning signs and severe dengue. The symptoms associated with these categories are summarized in Figure 1. Owing to the lack of antiviral drugs or vaccines for dengue, current treatments aim to alleviate the symptoms (Refs 2, 3). Therefore, clinicians must constantly monitor disease progression and apply appropriate treatment based on the patient's condition according to the guidelines provided by the WHO. Increased haematocrit concurrent with a rapid decline in platelet count, or thrombocytopenia, is one of the warning signs in dengue infection (Fig. 1). The level of thrombocytopenia may correlate with the severity of the disease (Refs 4, 5), and severe thrombocytopenia usually precedes the onset of the critical phase of the disease (Ref. 6). Additionally, severe thrombocytopenia in dengue patients could be the precursor to other severe dengue complications, such as plasma leakage and bleeding (Refs 5, 7). Dengue virus (DENV) has also been shown to activate platelets and other cells, causing the release of inflammatory cytokines that increase endothelium permeability (Ref. 8). Furthermore, DENV impairs platelet function, leading to endothelial dysfunction (Ref. 9). However, conflicting reports exist where haemorrhaging was not observed in some patients with severe thrombocytopenia (Ref. 10). Alterations in platelet level and activation status may also cause coagulation and fibrinolysis abnormalities, as reported here (Refs 9, 11). These findings signify the direct and indirect effects of platelet dysfunction in dengue. This review will focus on the mechanisms of thrombocytopenia during dengue infection and discuss a potential application based on this knowledge.

## Thrombocytes

Platelets, or thrombocytes, are colourless cell fragments produced by megakaryocytes through a process called thrombopoiesis. A normal platelet count ranges from 150 000 to 350 000 per microlitre of blood, but because of their small size, they make up just a tiny fraction of the blood volume (Ref. 12). Nonetheless, platelets are metabolically active and contain several functional organelles, including the endoplasmic reticulum, Golgi apparatus and mitochondria (Ref. 13). They also possess a wide range of surface receptors, adhesion molecules and granules (Ref. 14).

Platelet count is an important medical parameter, where a reduction in platelet count in the blood (thrombocytopenia) can be typically observed in numerous medical conditions such as viral infections, haematologic malignancies, autoimmune disorders and side effects of medications (Ref. 15). During dengue infection, the platelet count could drop to less than 30 000 per microlitre of blood (Refs 16, 17). A recent meta-analysis study found that thrombocytopenia is one of the two factors that could serve as independent predictive markers of severe dengue (Ref. 18).

Platelets are vital in blood coagulation to prevent blood loss during vessel injury. They play paramount roles in maintaining the structural integrity of the blood vessels, where the depletion of platelets has been shown to cause leakage because of microvessel disruption (Ref. 19). Platelets also safeguard and support the semi-permeability of the vessels by physically filling the gaps and secreting growth factors and cytokines to promote endothelial growth and maintain the barrier function of resting endothelium (Ref. 19). Conversely, activated platelets have been shown to secrete various inflammation mediators that can disrupt vessel integrity (Ref. 8). Thus, platelet level and activation status are critical in dengue progression, and aberrant platelet parameters may increase the risk of severe dengue.

## Mechanisms of thrombocytopenia in dengue infection

The mechanisms of dengue-mediated thrombocytopenia in patients still eluded researchers, but significant progress has been made in recent years. In short, dengue infection may disrupt platelet production in the bone marrow and/or expedite platelet clearance, causing thrombocytopenia. This review will be split into three main sections. In the first section (‘Dengue reduces platelet production’), we will focus on the effects of dengue infection on megakaryopoiesis and thrombopoiesis. In the second section (‘Dengue increases platelet activation and clearance’), we will discuss the effects of dengue infection on platelet activation and clearance. Lastly, we will discuss an application of the knowledge in this research area.

### Dengue reduces platelet production

#### Megakaryopoiesis and thrombopoiesis

Megakaryocytes are large haematopoietic cells ranging from 20 to 100 μm. The name ‘mega’ (large) – ‘karyo’ (nucleus) reflects their appearance, with a large and multilobulated nucleus that encompasses most of their granular cytoplasm. The nuclei of mature megakaryocytes are characterized as hyperploid, with an average ploidy of 16 N DNA, but could go up to 128 N (Ref. 20). Megakaryocytes are specialized cells that serve as the precursor in platelet biogenesis, where the granulated cytoplasm will pinch off, releasing up to 10^4^ platelets (Ref. 21).

Megakaryopoiesis, or the process of producing mature megakaryocytes, is orchestrated by several factors that play different roles at different stages of megakaryopoiesis. It begins with haematopoietic stem cells (HSCs) differentiating into common myeloid progenitors (CMPs). CMPs will then differentiate into megakaryocyte–erythrocyte progenitors (MEPs). MEPs will continue to differentiate along the megakaryocytic lineage to produce megakaryocyte progenitors (MKPs) or megakaryoblasts and eventually form mature megakaryocytes to produce platelets (Refs 22, 23).

Thrombopoietin (TPO) is a key factor that binds to the c-Mpl receptor to activate several signalling pathways involving Janus kinase, signal transducer and activator of transcription protein 3 and 5 (STAT3 and STAT5), p38 mitogen-activated protein kinase (p38 MAPK), extracellular signal-regulated kinase, phosphoinositide-3 kinase (PI3K) and protein kinase B (AKT) to initiate megakaryopoiesis (Ref. 24). Activation of these signalling pathways increases the expression of transcription factors such as GATA1, FOG1, FLI1, MYB and nuclear factor erythroid 2 (NF-E2), which upregulate genes crucial for megakaryocytes such as CD41, CD42b and CD61 (Refs 20, 24). TPO also increases the number of MKP cells, induces polyploidy and promotes megakaryocyte maturation (Ref. 24).

In addition to TPO, various cytokines such as the interleukin family (IL-1b, IL-3 and IL-6) work in synergy with TPO to promote the proliferation of MKP cells (Ref. 20). IL-1b has also been shown to upregulate the gene and protein levels of TPO, c-Jun, c-Fos, GATA-1 and NF-E2 in a dose-dependent manner, which likely serves as the basis for thrombocytosis during inflammation (Ref. 25).

Some cytokines and factors influence megakaryopoiesis independent of TPO-mediated signalling. For example, IL-1a induces proplatelet shedding into the bone marrow during megakaryocyte maturation (Ref. 20). C-C motif ligand 5 (CCL5) or regulated on activation, normal T cell expressed and secreted chemokine (RANTES) can also increase megakaryocyte ploidy and platelet production through CCR5 signalling (Ref. 26). A study has identified an enzyme, tyrosyl-tRNA synthetase variant (YRSACT), that can directly convert HSCs into MKPs to enhance megakaryopoiesis and thrombopoiesis of human induced-pluripotent cells that lack TPO signalling in vitro and in vivo (Ref. 27).

Thrombopoiesis is the process of platelet formation and is highly dependent on the development of mature megakaryocytes during megakaryopoiesis (Ref. 28). During this process, cytoskeletal proteins, membrane and granulated cytoplasm of mature megakaryocytes undergo extensive remodelling and form pseudopodial projections called proplatelets (Ref. 22). The proplatelets, characterized by long, thin shafts with swelling at the tip, are then released into the blood vessels (Ref. 29). The production of proplatelets requires intracellular factors such as the NF-E2 transcription factor and extracellular factors such as estradiol (Ref. 30) and shear force (Ref. 31). TPO, in addition to its central role in megakaryopoiesis, is also a crucial factor in thrombopoiesis (Ref. 24). As previously mentioned, TPO-mediated signalling elevates the level of the NF-E2 transcription factor, which is paramount in megakaryocyte maturation (Ref. 20). NF-E2 is also paramount in thrombopoiesis, as knocking out this factor resulted in severe thrombocytopenia because of impaired thrombopoiesis but no effects on megakaryopoiesis, as the megakaryocytes in these mice, though morphologically comparable with normal megakaryocytes, were unable to generate proplatelet extensions (Ref. 32). Thrombopoiesis and its critical factors and regulators have been extensively reviewed elsewhere (Refs 20, 33, 34).

#### Dengue impairs megakaryopoiesis and thrombopoiesis

Several viruses can disrupt megakaryocyte proliferation and maturation. Herpesviruses such as human cytomegalovirus and human herpes simplex virus impair megakaryopoiesis by causing apoptosis (Ref. 35). Pathogenic Hantaan orthohantavirus has been reported to infect and replicate in mature megakaryocytes by hijacking CD61 surface protein (Ref. 36).

DENV causes platelet reduction in patients, beginning typically on day 2 before the onset of the critical phase and persisting until days 6–7 (Ref. 1). One mechanism of thrombocytopenia is megakaryocyte infection and death. DENV has been shown to efficiently infect human megakaryocyte cell lines, primary human megakaryocytes or progenitors and megakaryocytes in humanized mice (Refs 37, 38, 39).

MEG-01 is one of the most common megakaryoblastic leukaemia cell lines in thrombocyte studies. Lahon *et al*. reported an effective DENV infection and replication in TPO-treated MEG-01 cells, compromising the PI3K/AKT/mTOR pathway that is essential in megakaryocyte survival and maturation (Ref. 39). DENV infection also caused significant cell death and repressed the expression of megakaryopoiesis-related transcription factors, namely GATA-1, GATA-2, NF-E2 and mature megakaryocyte marker CD61 (Ref. 39). Another study verified the susceptibility of MEG-01 cells to DENV infection, but reported a reduced DENV replication in phorbol-12-myristate-13-acetate (PMA) pre-treated MEG-01 cells when compared with control and MEG-01 cells treated with PMA after DENV infection (Ref. 40). In this study, Banerjee *et al*. showed that viral RNA copy number was significantly low in PMA-pre-treated cells at 2 dpi compared with control and PMA treatment post-infection samples. The authors stated that these PMA-differentiated MEG-01 cells were refractory against DENV infection/replication (Ref. 40). However, this statement contradicts several reports showing high DENV infection in mature megakaryocytes (Refs 37, 38, 41). A plausible explanation for the lower DENV replication in PMA-pre-treated cells could be because of PMA activity that activates protein kinase C (PKC), which in turn, inhibits DENV NS5 and suppresses DENV replication (Ref. 42). In contrast, PMA treatment post-DENV infection substantially increased DENV replication in MEG-01 cells (Refs 40, 43). Similar observations were reported in K562 cells treated with PMA after DENV infection (Ref. 43). PMA treatment post-DENV infection significantly increased DENV replication and infectious progeny production without influencing viral entry. In this study, PMA suppressed cellular reactive oxygen species (ROS) production by upregulating antioxidant NFE2L2 transcription factor to allow virus replication (Ref. 43). It is also possible that the maturation process with highly active transcriptional and translational machineries inadvertently create an intracellular milieu suitable for viral replication and virion production (Ref. 44).

In addition to cell lines, primary CD34^+^ MKPs obtained from umbilical cord blood are also prone to DENV infection, leading to disrupted colony formation and elevated apoptosis (Ref. 45). Similarly, another study reported that umbilical cord blood cells were highly susceptible to DENV infection, and these CD34^+^ cells serve as the reservoir of viral progenies (Ref. 46). Interestingly, these progenies were mostly latent.

In a humanized mouse model, a higher percentage of DENV infection in mature CD41a^+/−^ + CD42b^+^ megakaryocytes (35%) was observed when compared with immature CD41a^+^ + CD42b^−^ megakaryocytes (1.5%) (Ref. 37). However, the number of mature human megakaryocytes in this model was greatly diminished (Ref. 37). Nevertheless, this finding is consistent with another study where DENV displayed a selective tropism for CD42-expressing MEG-01 cells compared with CD42^−^ or CD41^+^ cells (Ref. 41). Another study, using patient samples and rhesus monkeys, showed CD61+ cells were susceptible to DENV infection (Ref. 38).

In addition to impaired megakaryopoiesis, DENV infection can hamper thrombopoiesis by significantly reducing proplatelet formation in PMA-treated MEG-01 cells (Ref. 40). The exact mechanism of this effect is currently unclear but may involve the NF-E2 transcription factor, a crucial thrombopoiesis factor. DENV infection markedly reduced NF-E2 protein expression in mature MEG-01 cells (Ref. 39). Another potential mechanism may involve the sphingosine 1-phosphate receptor (S1pr). S1pr1, one of the sphingosine-1-phosphate receptor subtypes, has been studied in dengue infection settings but mostly on its roles in regulating vascular permeability where sphingosine 1-phosphate (S1P) levels were found markedly reduced in acute dengue patients (Ref. 47). S1pr1 was reported to be crucial in platelet production, where knocking out of S1pr1 in mice resulted in severe thrombocytopenia concomitant with significant reductions in proplatelet formation and proplatelet shedding (Ref. 48). Thus, the S1P–S1pr1 axis may also be implicated in dengue-mediated thrombopoiesis impairment.

A study unravelled a novel role of DENV NS3 protease in cleaving GrpE protein homologue 1 (GrpEL1), a cochaperone of mitochondrial heat shock protein 70 (mtHsp70) (Ref. 49). GrpEL1 and its orthologue, GrpEL2, form a subcomplex that is pivotal in maintaining the stability of nucleotide exchange factor for mtHsp70, thus modulating critical mtHsp70 functions such as importing mitochondrial pre-protein into the matrix (Ref. 50). Overexpression of NS3 protease reduced GrpEL1 protein level in vitro. More importantly, GrpEL1 protein level was significantly reduced in dengue patient samples, especially in dengue haemorrhagic fever (DHF) and dengue shock syndrome (DSS) patients, which correlated with thrombocytopenia level in these patients (Ref. 49). Reduced GrpEL1 compromised mitochondria health and functions, leading to thrombocytopenia (Ref. 51). Another form of DENV protease, NS2B-NS3 protease, which localized to the nucleus, could cleave erythroid differentiation regulatory factor 1 (EDRF1) transcription factor (Ref. 51). EDRF1 is critical in platelet formation as it regulates the levels of GATA1 and spectrin, a cytoskeletal protein essential for membrane re-organization during platelet biogenesis (Ref. 52). Overexpression of NS2B-NS3 protease significantly diminished EDRF1 and GATA1 protein levels. Similarly, EDRF1 level was also reduced in dengue patients, especially in thrombocytopenic dengue haemorrhage patients and dengue shock syndrome patients (Ref. 51). Thus, NS3 contributes to thrombocytopenia by cleaving EDRF1 to impair thrombopoiesis, and cleaving GrpEL1 to cause mitochondrial dysfunction and disrupt platelet formation.

In short, DENV can infect megakaryocytes at different stages of cell maturation and impair platelet production, leading to reduced platelet formation.

### Dengue increases platelet activation and clearance

#### Platelet activation

The roles of platelets during haemostasis and thrombosis events begin with a series of activation processes that include platelet adhesion, network extension and thrombus formation involving multiple factors, as summarized in Table 1 (Ref. 53). Briefly, in the event of high shear or vascular injury, nearby platelets will be exposed to von Willebrand factor (vWF) on the vessel wall (Ref. 54). The vWF will serve as a bridge to connect platelet glycoprotein (GP) Ib receptor complex on the platelet's membrane with the collagen layer, allowing platelet GPVI receptors to adhere directly onto the collagen matrix for a strong adhesion (Ref. 55). The vWF also facilitates the activation of circulating platelets by connecting the GP IIb/IIIa receptor on the activated platelets to the GP Ib on the circulating platelets to activate them. Bound fibrinogen will strengthen the crosslink of the GP IIb/IIIa receptor on both activated platelets (Ref. 56). The activated platelets undergo structural changes with the formation of pseudopodia and initiate the release of platelet granules (Ref. 56). The released granules contain chemokines and cytokines to signal other circulating platelets to interact with the adhering platelets and form extensions of activated platelet network (Ref. 57).

**Table 1.** Factors in platelet activation and aggregation

Factor	Signalling receptors on platelets	Functions	Expression or activity during dengue infection
Extracellular matrix molecule (ECM)			
Collagen vWF	GPIb GPIIb/GPIIIa GPVI	Platelet adhesion Activation of GPIIb/GPIIIa Release of ADP and TxA2 Promote structural changes Platelet aggregation	NS1 with suboptimal collagen causes platelet aggregation (Ref. 58) vWF is highly elevated (Ref. 59) Higher vWF binding to platelet in critical and acute patients (Ref. 60)
Granule secretory products			
ADP TxA2 Chemokines Serotonin	P2Y_1_ and P2Y_12_ TP Chemokine and cytokine receptors 5HT-2A	Recruitment of circulating platelet Induce the release of TxA2 Platelet recruitment Promote the formation of thrombus Induce platelets granule secretion Platelet adhesion Platelet aggregation	NS1 with suboptimal ADP causes platelet aggregation (Ref. 58) TxA2 increases in IgM+ patients with mild symptoms (Ref. 61) Different cytokine/chemokine profiles for DwWS/SD versus DwoWS and healthy controls (Ref. 62) Mast cell-derived serotonin was elevated in DENV-infected WT mice (Ref. 63)
Others			
Prothrombin Thrombin	PAR-1 and PAR-4	Platelet aggregation Activate fibrinogen to fibrin Formation of platelet plug	High magnitude of prolonged aPTT and PT in patients in a meta-analysis study (Ref. 11)
Platelet-leucocyte stimuli	P-selectin	Platelet adhesion Platelet-leucocyte aggregate formation	Elevated (Refs 16, 58, 64)

Platelets contain *α*- and dense granules. *α*-Granules are the most abundant secretory organelles in platelets, and they contain adhesive proteins, cytokines and chemokines crucial for platelet adhesiveness and thrombus formation (Ref. 65). These chemokines such as RANTES, CXCL1, platelets factor 4 (PF4 or CXCL4) and IL-6 interact with platelet surface receptors and immune cells (Ref. 66). RANTES (CCL5) binds to chemokine receptors CCR1, CCR3 and CCR5 on platelets to stimulate other platelets and recruit monocyte to the inflamed endothelium (Ref. 67). PF4 also facilitates platelet adhesion and mediates leucocyte interaction (Ref. 68).

Dense granules, on the other hand, contain small molecules such as adenosine diphosphate (ADP), adenosine triphosphate (ATP), calcium ions serotonin and phosphates (Ref. 65). ADP is crucial in platelet activation through its interaction with two G-protein-coupled receptors (P2Y1 and P2Y12) to initiate platelet aggregation and provide a feedback mechanism to increase the secretion of thromboxane A2 (TxA2) and other agonists (Ref. 69). Studies showed that the absence of P2Y12 in humans resulted in haemorrhage, and lacking P2Y1 in mice prolonged the bleeding time (Ref. 70). In addition to procoagulant activity, ADP also mediates the release of TxA2 where the interaction of TxA2 with TxA2 receptor (TP) attracts other platelets to bind to the adherent platelets, forming a stable thrombus (Ref. 56). Thrombin plays essential roles in plug formation through protease-activated receptor 1 and 4 (PAR-1 and PAR-4) that are coupled to Gq and G12/G13 proteins, respectively (Ref. 71). In addition, hormones such as serotonin activate platelets through 5-hydroxytryptamine 2A (5HT-2A) receptors and promote platelet aggregation (Ref. 72).

#### Platelet death and clearance

Platelets generally have a life span of 5–10 days before clearance from the body (Ref. 73) via several mechanisms such as platelet apoptosis, antibody-mediated phagocytosis in the spleen, removal by the Kupffer cells in the liver via lectin-glycan recognition or massive platelet release because of blood loss (Refs 74, 75). Ageing platelets express a higher level of phosphatidylserine (PS), a type of phospholipid that activates apoptosis (Ref. 76). Other pro- and anti-apoptotic factors such as Bak, Bax and Bcl-XL are also involved in platelet apoptosis (Ref. 75). Thrombin-activated platelets undergo metabolic exhaustion marked by mitochondrial depolarization, accumulation of ROS and ATP depletion, followed by platelet dysfunctional and disintegration, calpain-activation and eventually platelet fragmentation (death) (Ref. 77). Activated and apoptotic platelets are also exposed to clearance by macrophages. Desialylation of platelet via neuraminidase or vWF action induces platelet clearance and acute thrombocytopenia (Ref. 75). Platelets are also cleared from the body through platelet plug clearance (Ref. 16).

#### Dengue increases platelet activation and apoptosis

Invading pathogens such as bacteria, parasites and viruses activates platelets via pattern recognition receptors such as Toll-like receptors (TLRs) and C-type lectin receptors (CLRs), complement Fc receptors (FcR), major histocompatibility complex (MHC) class I and many more (Refs 41, 78, 79). Excessive platelet activation and apoptosis, marked by elevated P-selectin and annexin V level, respectively, was observed in dengue patients during the febrile and defervescence phases, causing thrombocytopenia in these patients when compared with healthy controls, non-dengue febrile patients and non-thrombocytopenic dengue patients (Ref. 64). Similarly, increased platelet activation concurrent with thrombocytopenia was observed in dengue patients during the critical phase (days 4–6) (Ref. 16). Dengue-induced thrombocytopenia was replicated in vivo when mice injected with DENV2 suffered thrombocytopenia concomitant with elevated platelet activation (Ref. 63). Several mechanisms for platelet activation and death mediated by the DENV are summarized below.

*DENV NS1*. Following infection, DENV hijacks the translational machinery to produce new dengue proteins such as dengue non-structural 1 (NS1) protein and new virions. NS1 binding to TLR4 on primary human platelets enhanced platelet activation marked by enhanced P-selectin expression, causing platelet apoptosis (Ref. 58) and the released of granule-stored chemokines such as RANTES, macrophage migration inhibitory factor (MIF) and PF4 (Ref. 80). Full-length NS1 and all NS1 domains, especially the wing domain, could activate platelets, but only the full-length NS1 could induce platelet aggregation in the presence of platelet agonists (Ref. 81). The increased P-selectin expression on NS1-activated platelets promoted platelet adherence to endothelial cells and macrophages, compromising endothelial permeability and increasing platelet phagocytosis, respectively (Ref. 58). DENV NS1 also amplified thrombo-inflammatory responses and induced the synthesis of pro-IL-1*β* but not its secretion (Ref. 80). IL-1*β* secretion from NS1-activated platelets was mediated by nucleotide-binding domain leucine-rich repeat-containing protein (NLRP3)-caspase-1 inflammasome (Ref. 80). More importantly, the secretion of pro-inflammatory cytokines such as IL-1*β* and MIF contributed to vascular permeability and tissue injury (Ref. 82). DENV NS1 also sensitized the platelets to aggregation where exposure to DENV NS1 alone could not stimulate platelet aggregation, but co-treatment of NS1 with subthreshold levels of platelet agonists markedly increased platelet aggregation which contributed to thrombocytopenia (Refs 58, 81).

*DENV envelope protein*. Dengue can infect cells via two CLRs, specifically DC-SIGN (dendritic cell-specific intercellular adhesion molecule-3-grabbing nonintegrin) and CLEC-2 (C-type lectin-like-receptor). The N-glycans on the DENV envelope protein can bind to DC-SIGN, causing virus adsorption onto the plasma membrane (Ref. 83). The binding of DENV to DC-SIGN was shown to increase platelet activation, cause mitochondria dysfunction and trigger caspase-dependent apoptosis (Ref. 64).

As previously mentioned, co-stimulation of NS1-activated platelet with suboptimal ATP concentration upregulated IL-1*β* secretion, possibly through NLRP3 inflammasome (Ref. 80). Similarly, DENV envelope domain III (EIII) also activated platelet, and caused thrombocytopenia in vivo primarily through NLRP3-mediated pyroptosis (Ref. 84). EIII also impaired clotting time, and interestingly, inhibition of this response pathway via NLRP3 inhibition or EIII blocking could significantly improve the conditions (Ref. 84).

*Other factors*. Proteomics analysis comparing platelets from dengue patients with healthy controls showed 167 differentially abundant proteins, with most of the proteins involved in ‘antigen processing and presentation’ and ‘platelet activation’ processes (Ref. 85). As expected, P-selectin, PF4 and RANTES were among the highly upregulated factors in dengue (Ref. 85). Histones H2A and HLA class I were also upregulated in dengue-infected platelets. The binding of circulating histone H2A to the TLR4 receptor on platelets caused platelet activation that could be completely blocked by anti-histone H2A monoclonal antibody (Ref. 85).

CLEC-2 also plays a pivotal role in platelet activation. Elevated CD62p and CD63 expression after DENV infection was abolished in human platelets treated with CLEC2-mAb and clec2^−/−^ mouse platelets (Ref. 86). More importantly, the study reported that activated platelets via CLEC2 produced extracellular vesicles that activated neutrophils and macrophages via CLEC5A and TLR2 and contributed to the formation of neutrophil extracellular traps (NETs). NET level in the plasma was reported to inversely correlate with platelet count and positively correlate with P-selectin expression during days 7–13 of disease in acute dengue patients (Ref. 87).

Mast cell-derived serotonin was reported to be elevated during dengue infection in vitro and in vivo and activated the platelets through the 5HT-2 receptor, which was then demonstrated to be phagocytosed (Ref. 63). Inhibiting the 5HT-2 receptor reduced dengue-induced thrombocytopenia and platelet activation (Ref. 63). However, although the roles of serotonin in platelet activation, aggregation and phagocytosis were sufficiently established in vivo (Ref. 63), an earlier metabolomic study showed a significant reduction of serotonin in dengue high fever patients compared with dengue fever patients, especially during the febrile and defervescence phases (Ref. 88). The study also proposed that reduced serotonin coupled with elevated interferon gamma may serve as a biomarker of severe dengue. The conflicting reports on serotonin levels post-dengue infection could be because of the different hosts where, as opposed to rodents, mast cells are not the primary source of serotonin in humans (Ref. 89). Thus, the role of serotonin in dengue and dengue-mediated thrombocytopenia requires further investigation.

Another important mechanism of platelet activation is through antibody-dependent enhancement or ADE. This mechanism involves the interaction between virus-immunoglobulin (Ig) G complexes and FcR-bearing cells (Ref. 90). Unlike other factors, ADE is primarily active during secondary DENV infection as the IgG antibody secreted during the first infection binds to the second-generation virus and permits faster and more severe infections via FcR expressed on immune cells and platelets (Ref. 91). A previous study showed that immature dengue virion becomes highly infectious in the presence of anti-prM antibodies that facilitate viral entry and enhance the intracellular processing of prM to M protein (Ref. 92). Another study demonstrated that DHF/DSS patients showed elevated afucosylated IgGs with strong affinities towards Fc*γ*RIIA and Fc*γ*RIIIA receptors expressed on the platelets, leading to platelet reduction. The level of afucosylated IgGs correlated with thrombocytopenia severity in these patients and was a significant risk factor for thrombocytopenia (Ref. 93). As FcRs are expressed on immune cells, it was postulated that thrombocytopenia observed in this study could be because of enhance platelet activation, platelet sequestration and/or antibody-dependent cell cytotoxicity (Ref. 93). Additionally, afucosylated IgGs could also serve as a prognostic factor of dengue disease severity (Ref. 94).

#### Dengue increases platelet clearance

The activated and apoptotic platelets are subsequently cleared from the body in several ways. Phagocytosis of apoptotic platelets by macrophages in acute and early convalescence dengue patients was reported to be 2.5–3.5 times higher than the platelets from healthy controls (Ref. 95). Phagocytosis was substantially reduced when these patient-derived platelets were pre-treated with D89E mutant protein that masked the PS on the apoptotic platelets (Ref. 95). Phagocytosis of DENV2-activated platelets by primary human monocytes was also reported in vitro (Ref. 16).

The vWF could also mediate platelet clearance. vWF, elevated in thrombocytopenic dengue patients, increased platelet desialylation, thus exposing the platelet to clearance via Ashwell–Morell receptor-mediated pathway (Ref. 60). DENV-activated platelets also showed high levels of bound complement factor C3 and IgG, suggesting elevated platelet lysis, clearance and increased thrombus formation that caused platelet depletion in the plasma (Refs 16, 93). Immune-mediated platelet clearance and lysis during dengue infection have been reviewed here (Ref. 96).

**Figure 2.**
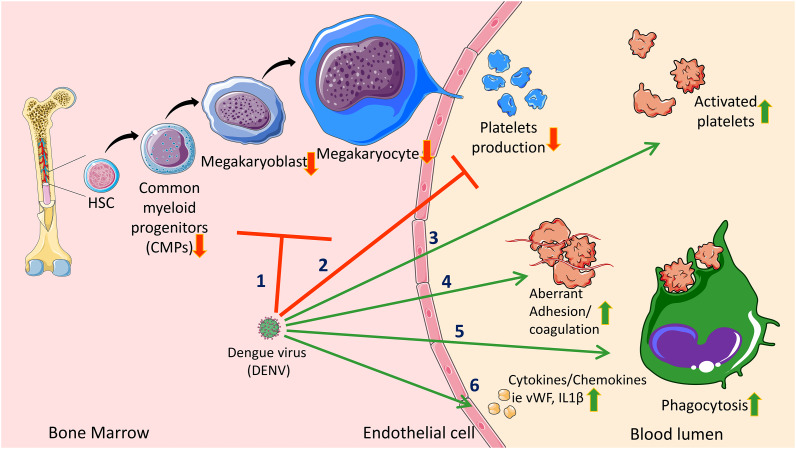
Summary of DENV-mediated thrombocytopenia. DENV causes thrombocytopenia in several ways. (1) DENV impairs megakaryopoiesis. DENV infects and causes apoptosis of megakaryocytes and the progenitor cells. DENV also prevents megakaryocyte maturation. (2) DENV impairs thrombopoiesis by reducing megakaryocytes and interfering with platelet formation. (3) Secondary DENV infection causes elevated platelet activation via IgG–FcR connection in the ADE process. (4) DENV increases platelet activation. (5) DENV causes cytokines, chemokines and other factors to be released from infected platelets and nearby endothelial cells. (6) DENV infects and causes platelet apoptosis. (7) ADE, elevated platelet activation and secretion of various factors cause the platelets to coagulate. (8) Coagulated platelets and apoptotic platelets are cleared from the circulation by phagocytes. Regular arrows (→) in green indicate stimulatory modifications. Blunt-ended arrows (˧) in red indicate inhibitory modifications.

The events and processes described in the sections ‘Dengue reduces platelet production’ and ‘Dengue increases platelet activation and clearance’ are not mutually exclusive and may simultaneously occur to exacerbate dengue symptoms. Figure 2 summarizes these events. Since the pathophysiology and progression of dengue fever vary among patients, it is challenging to determine the predominant mechanism for thrombocytopenia in patients. Nonetheless, research in this field has improved disease management. An emerging application of knowledge in this field is immature platelet fraction (IPF), which will be briefly discussed in the next section.

## Potential application: IPF% as a predictive tool for platelet recovery in dengue patient

Thus far, there is no treatment against dengue, but targeting the molecular factors involved in dengue-induced thrombocytopenia could prevent or reverse this complication, and could be vital in managing dengue progression to severe dengue (Refs 63, 84, 86). There have been considerable advances in platelet study that could be applied to address platelet abnormality in dengue.

IPF is an automated measurement of reticulated platelet levels. These reticulated platelets are nascent platelets with punctate and coarse condensations. They still contain the mRNA that can be stained with nucleic acid binding dyes and quantified (Ref. 97). Essentially, this parameter reflects the rate of thrombopoiesis that is upregulated in response to elevated platelet consumption or clearance (Ref. 97).

Normal IPF% is in the range of 1.1–6.1%, with a mean of 3.4%, but in hyperdestructive thrombocytopenic patients, the IPF% could elevate to 22.3% in autoimmune thrombocytopenic purpura patients and 17.2% in thrombotic thrombocytopenic purpura patients (Ref. 97). In patients with hypoproductive conditions such as aplastic anaemia and chemotherapy-induced bone marrow toxicity, IPF% levels remain unchanged, corroborating the utility of this parameter in evaluating thrombopoiesis activity (Ref. 98). In dengue patients, IPF% increases to varying degrees (Refs 99, 100).

In dengue, IPF may serve as an indicator of platelet recovery from dengue-induced thrombocytopenia. In a study conducted on 32 dengue patients, more than 84% of the patients showed platelet count recovery 24 h after reaching the IPF peak (Ref. 99). Similarly, another study showed an inverse correlation between IPF% and platelet count where IPF% was high and platelet count was low during the early phase of the disease (days 2–9). Platelet count rebounded 24–48 h after peak IPF% (Ref. 100). This inverse correlation was less pronounced in severe dengue patients, where platelet recovery was slightly delayed (Ref. 100).

Another important observation was severe dengue patients had a significant elevation of IPF% on days 3–5 after the onset of fever compared with non-severe dengue patients (Ref. 100). Another study reported a similar trend where severe dengue patients had higher IPF% when compared with non-severe dengue patients (Ref. 101). The observed changes in this parameter suggest that platelet consumption or clearance could be the primary underlying mechanism of dengue-induced thrombocytopenia.

In short, IPF% may serve as a useful indicator for platelet recovery as this parameter signals the body's response to platelet death and clearance because of DENV infection. Platelet recovery is typically observed within 24–48 h post-peak IPF% in most dengue patients. Thus, this parameter may aid clinicians in monitoring the progress of the disease. Nonetheless, further studies are needed to determine the utility of this parameter in dengue management as well as to verify the correlation between IPF% and dengue severity.

## Conclusion

In conclusion, the DENV can infect megakaryocytes and their progenitor cells, causing cell death to reduce the rate of platelet production. DENV also induces platelet activation, leading to platelet apoptosis, clearance and phagocytosis. These non-mutually exclusive events complicate the efforts to prevent or treat this complication in dengue. However, there has been significant progress in understanding and treating thrombocytopenia, which will benefit dengue patients. IPF, for example, could be further investigated as a prognostic tool to determine platelet recovery in dengue patients.
